# Forest *Saccharomyces paradoxus* are robust to seasonal biotic and abiotic changes

**DOI:** 10.1002/ece3.7515

**Published:** 2021-04-07

**Authors:** Primrose J. Boynton, Dominika Wloch‐Salamon, Doreen Landermann, Eva H. Stukenbrock

**Affiliations:** ^1^ Biology Department Wheaton College Norton MA USA; ^2^ Environmental Genomics Research Group Max‐Planck Institute for Evolutionary Biology Plön Germany; ^3^ Faculty of Biology Institute of Environmental Sciences Jagiellonian University Kraków Poland; ^4^ Botanical Institute Christian‐Albrechts Universität Kiel Germany

**Keywords:** environmental yeast, killer yeast, microbial ecology, rapid adaptation, selection

## Abstract

Microorganisms are famous for adapting quickly to new environments. However, most evidence for rapid microbial adaptation comes from laboratory experiments or domesticated environments, and it is unclear how rates of adaptation scale from human‐influenced environments to the great diversity of wild microorganisms. We examined potential monthly‐scale selective pressures in the model forest yeast *Saccharomyces paradoxus*. Contrary to expectations of seasonal adaptation, the *S. paradoxus* population was stable over four seasons in the face of abiotic and biotic environmental changes. While the *S. paradoxus* population was diverse, including 41 unique genotypes among 192 sampled isolates, there was no correlation between *S. paradoxus* genotypes and seasonal environments. Consistent with observations from other *S. paradoxus* populations, the forest population was highly clonal and inbred. This lack of recombination, paired with population stability, implies that selection is not acting on the forest *S. paradoxus* population on a seasonal timescale. *Saccharomyces paradoxus* may instead have evolved generalism or phenotypic plasticity with regard to seasonal environmental changes long ago. Similarly, while the forest population included diversity among phenotypes related to intraspecific interference competition, there was no evidence for active coevolution among these phenotypes. At least ten percent of the forest *S. paradoxus* individuals produced “killer toxins,” which kill sensitive *Saccharomyces* cells, but the presence of a toxin‐producing isolate did not predict resistance to the toxin among nearby isolates. How forest yeasts acclimate to changing environments remains an open question, and future studies should investigate the physiological responses that allow microbial cells to cope with environmental fluctuations in their native habitats.

## Graphical abstract

We have sampled populations of the wild yeast species *Saccharomyces paradoxus* in a natural forest to study spatial and temporal variation. The distribution of genetic and phenotypic diversity was analyzed using microsatellite markers and in vitro assays. We show that yeast populations are characterized by stability, high dispersal potential, clonality, and inbreeding, while still including genetic and phenotypic diversity.

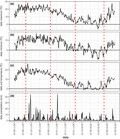

## 1 INTRODUCTION

All organisms, large and small, live in changing environments. These changing environments are diverse: Environmental changes have frequencies ranging from fast to slow; are directional, random, or cyclic; and come from abiotic and biotic sources (Andrews & Brasier, 2005; Kidwell, 2015; Meyers & Bull, 2002). Organisms’ responses to changes are equally diverse: They can evolve to adapt to new environments; acclimate through temporary changes in physiology or behavior; adjust their ranges; or respond through a combination of these and other processes (Nadeau & Urban, 2019). Microbial responses to changing environments are emerging as an important theme of current research, especially as researchers grapple with human impacts on climate and biodiversity (Antwis et al., 2017). For example, responses to climate change among more easily studied macroorganisms are varied and difficult to extend to microorganisms (Rowe et al., 2015; Smith et al., 2019).

Most information about microbial responses to environmental changes comes from human‐influenced environments, in which rapid adaptation is common. Rapid adaptation is frequently observed in laboratory experimental evolution: Under controlled laboratory conditions, microbial fitness in new environments increases substantially over timescales of days to months (Gómez & Buckling, 2011; Luria & Delbrück, 1943; Rafaluk‐Mohr et al., 2018; Rainey & Travisano, 1998). For example, populations of the model domesticated yeast *Saccharomyces cerevisiae* experienced significantly increased fitness after only tens of generations, or a few weeks, in a novel chemostat environment (Goddard et al., 2005). Similarly, rapid microbial adaptation is well documented in other human‐influenced systems, including antibiotic‐treated patients in hospitals, crop systems with microbial pathogens, domesticated fermentations, and the human microbiome (Beaume et al., 2017; Davies & Davies, 2010; McDonald & Stukenbrock, 2016; Verstrepen et al., 2003). Observations of rapid adaptation in human‐influenced microbial populations may or may not extend to wild microbial populations because humans influence the ecology of their associated microbial populations (Gibbons & Rinker, 2015). For example, humans generally interact with rapidly multiplying microbial populations under low dispersal limitation (Beggs et al., 2015; Kawecki et al., 2012). In addition, laboratory experimental evolution studies often impose strong selective pressures under controlled conditions (Swamy & Zhou, 2019), but the selective pressures relevant in nature are often unknown and likely to be complex. Studies of microbial diversity and fitness *in realistic natural environments* are needed to understand microbial responses to their changing native habitats.

Populations of *Saccharomyces paradoxus*, the wild sister species of *S. cerevisiae*, are an ideal model system for investigating adaptive evolution in nature. Both *S. paradoxus* and *S. cerevisiae* evolve quickly in laboratory evolution experiments (Goddard & Bradford, 2003; Goddard et al., 2005; Ratcliff et al., 2012; Selmecki et al., 2015; Wloch‐Salamon et al., 2017). Unlike *S. cerevisiae, S. paradoxus* has never been domesticated; it lives in forest environments, primarily in the northern hemisphere (Boynton & Greig, 2014; Robinson et al., 2016). It associates with oak trees and can be found on bark, exudates, leaf litter, and soil near oaks (Boynton et al., 2019; Kowallik & Greig, 2016; Kowallik et al., 2015; Naumov et al., 1998; Sniegowski et al., 2002). While the range of *S. paradoxus* is limited by maximum summer temperature, population sizes can be stable year‐round through seasonal changes in temperature, moisture, nutrient availability, and the activity of other organisms (Kowallik & Greig, 2016; Robinson et al., 2016; Voříšková et al., 2014). In our study forest in northern Germany, the number of *S. paradoxus* cells per gram of leaf litter does not vary from season to season, suggesting that *S. paradoxus* dormancy does not follow seasonal cycles, and that cells are instead as active in winter as they are in summer (Kowallik & Greig, 2016).

Seasonal abiotic changes, and especially temperature changes, are a plausible selective pressure on forest *S. paradoxus*. Temperature is an important selective pressure across *Saccharomyces* species: Different species have different temperature optima, and environmental temperatures determine *Saccharomyces* species ranges (Robinson et al., 2016; Salvadó et al., 2011; Sampaio & Gonçalves, 2008; Sweeney et al., 2004). In laboratory experimental evolution, *S. cerevisiae* adapts to previously lethal temperatures after a few days (Huang et al., 2018); this rapid laboratory adaptation suggests that temperature adaptation is possible on seasonal timescales in wild *Saccharomyces* species. Rapid adaptation to seasonal changes has also previously been observed in wild animal populations, which have longer generation times than laboratory *Saccharomyces*. For example, differences in fitness among Leopard Frog morphs between winter and summer lead to seasonal differences in allele frequencies (Merrell & Rodell, 1968), and stress‐tolerant *Drosophila* genotypes are more common in the spring (immediately after winter) than in the fall (immediately after summer) (Behrman et al., 2015). Seasonal environmental changes in *S. paradoxus* habitats are more complex than the changes in temperature investigated in laboratory experimental evolution; we investigated whether a *S. paradoxus* population changed in response to these complex seasonal environmental changes.

As with abiotic environmental changes, biotic interactions can select organisms over short timescales. For example, laboratory host–pathogen and predator–prey coevolution experiments often result in rapid coadaptation among organisms as diverse as animals, bacteria, and algae. Nematodes adapt to pathogenic bacteria over tens of generations by increasing sexual recombination even as their pathogens evolve greater infectivity (Morran et al., 2011), and green algae adapt their morphologies to rotifer predators over tens of days (Becks et al., 2010). Yeast species also rapidly adapt to biotic selective pressures during laboratory evolution. For example, bacterial communities select for diverse *S. cerevisiae* multicellular phenotypes after 3 months of laboratory evolution (Quintero‐Galvis et al., 2018). Bacteria also select for increased fermentation ability and temperature tolerance in *Lachancea* species (*Lachancea* is a genus of yeasts in the same family as *Saccharomyces*) after months of experimental evolution (Zhou et al., 2017, 2019). Intraspecific interactions may be equally important for adaptation, as demonstrated by the evolution of *S. cerevisiae* resistance to intraspecific toxins over days of laboratory evolution (Pieczynska et al., 2016).

Intraspecific interference competition, mediated by “killer toxins,” is a plausible biotic selective pressure on forest *S. paradoxus*. Killer toxins are secreted yeast toxins that kill nearby sensitive yeast cells, often of the same species; in members of the genus *Saccharomyces*, toxin production is often coded on cytoplasmic dsRNA viruses (Boynton, 2019; Magliani et al., 1997; Schmitt & Breinig, 2006). These toxins are well studied in *S. cerevisiae* and have been reported in *S. paradoxus* (Chang et al., 2015; Pieczynska et al., 2013). However, the primary effect of killer toxins in nature is unclear: These toxins may promote interference competition in nature, maintain viruses in host cells, mediate cell–cell communication, or have another, as yet undetermined, function (Boynton, 2019). Toxin production may also be influenced by the abiotic environment. Viruses coding for killer toxins are occasionally lost after heat treatments in the laboratory (Wickner, 1974); if heat also causes virus loss in natural environments, the killer phenotype may be more common in cooler months than in warmer months. Yeasts that produce killer toxins (“killer yeasts”) select for toxin resistance in sensitive yeasts after a few days, or tens of generations, of evolution in the laboratory (Pieczynska et al., 2016). If killer toxin‐mediated interference competition is an important selective pressure in nature, *S. paradoxus* populations may be under considerable pressure to evolve resistance to these toxins. If selection by killer toxins shapes forest *S. paradoxus* populations, we would expect selection for resistance to local killer toxins evolves rapidly, perhaps over seasonal timescales.

**FIGURE 1:**
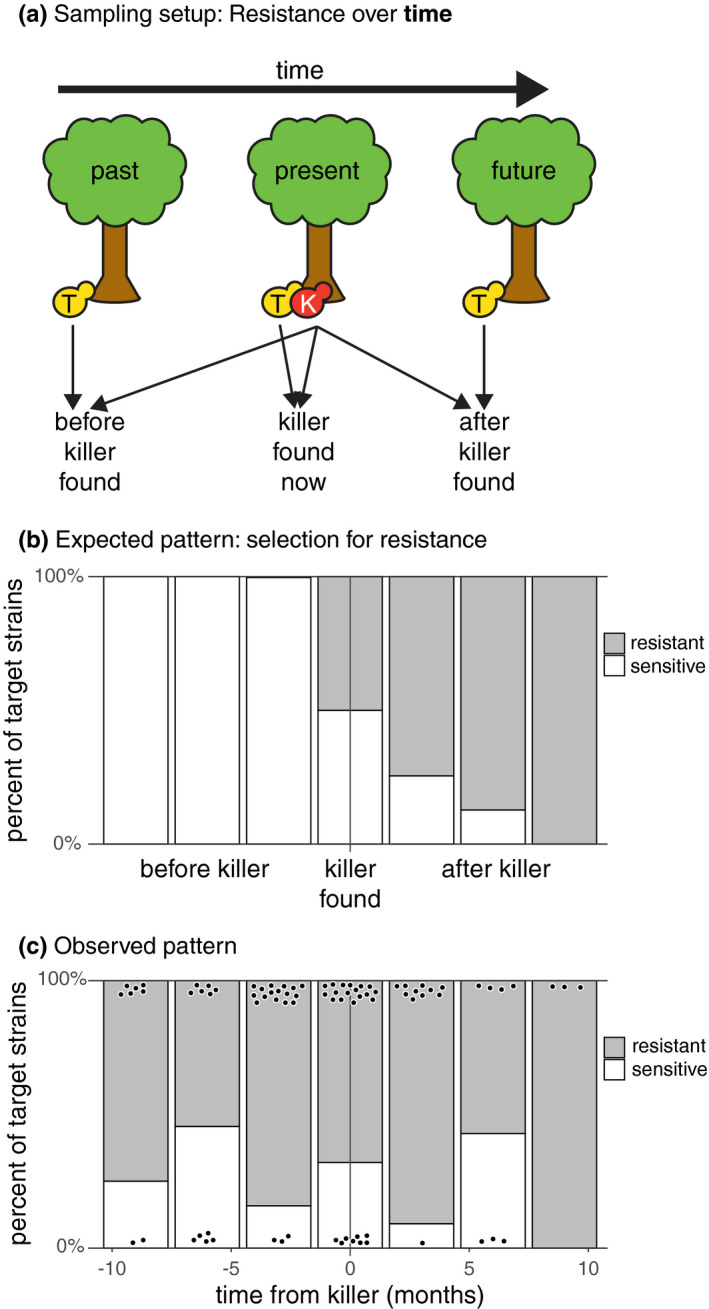
Resistance to killer toxins over time. (a) Schematic of experimental design. For each toxin‐producing killer strain (“K” in red), target strains (“T” in yellow) were selected from before, during, and after the killer strain was found, as available. Each target strain was tested for resistance or sensitivity to the killer strain. (b) Cartoon of expected results if the presence of a toxin‐producing strain selects for resistance over time. The *x*‐axis depicts the time in which each target yeast was found relative to the time in which its corresponding killer was found. The *y*‐axis depicts the expected frequencies of resistant strains before, while, and after a killer was found. (c) Observed proportion of resistant strains over time. Gray bars indicate proportion of resistant strains; white bars indicate proportion of sensitive strains; and black points indicate individual tested target strains

**FIGURE 2:**
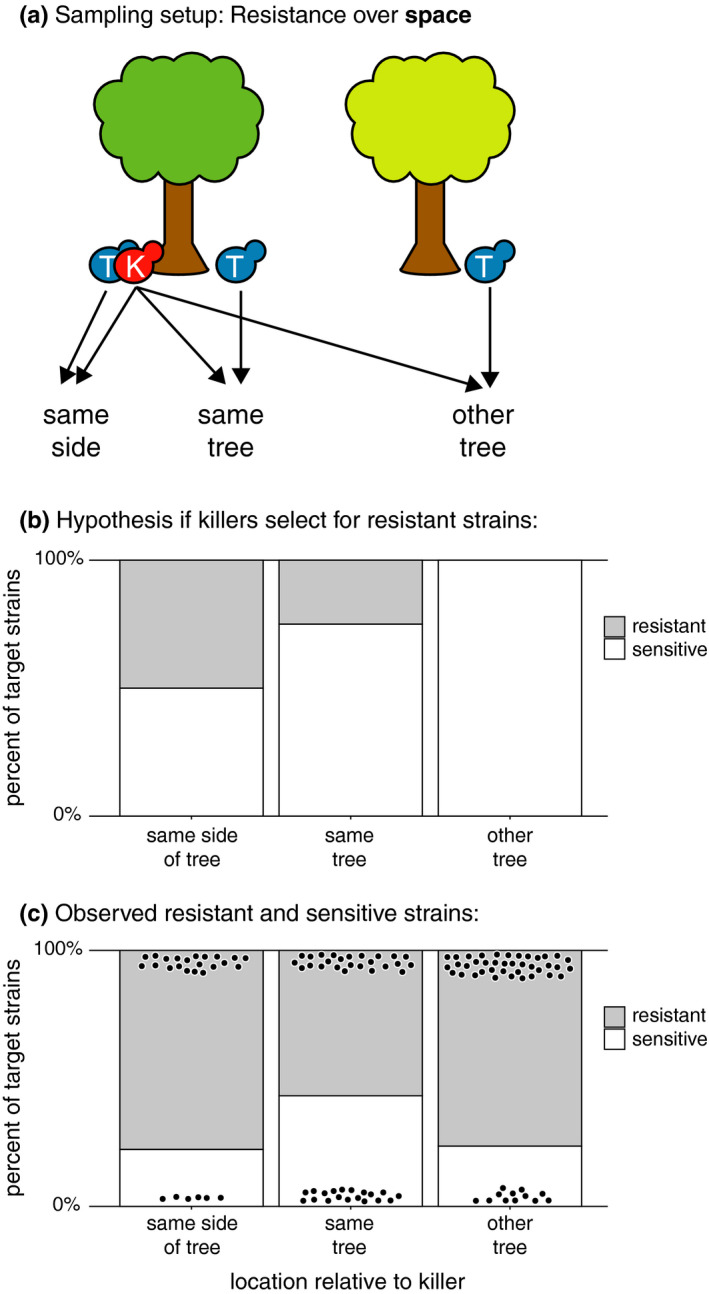
Resistance to killer toxins over space. (a) Schematic of experimental design. For each toxin‐producing killer strain (“K” in red), target strains (“T” in blue) were selected from the same location, another location at the same tree, and a randomly selected other sampled tree. Each target strain was tested for resistance or sensitivity to the killer strain. (b) Cartoon of expected results if the presence of a toxin‐producing strain selects for resistance over space. The *x*‐axis depicts the location from which each target yeast was found relative to its corresponding killer's location. The *y*‐axis depicts the expected frequencies of resistant strains before, while, and after a killer was found. (c) Observed proportion of resistant strains over space. Gray bars indicate proportion of resistant strains; white bars indicate proportion of sensitive strains; and black points indicate individual tested target strains

For this study, we investigated the potential for monthly‐scale adaptation to seasonal environmental changes and killer toxins among members of a *S. paradoxus* population from a German forest. We had previously determined that *S. paradoxus* isolates from this forest are genetically and phenotypically diverse (Boynton et al., 2019). For the current study, we measured the population structure and killer‐related phenotypes of a collection of 192 *S. paradoxus* isolates, collected over four seasons, to understand whether seasonal environments correlate with population structure and whether coadaptation among isolates evolves over seasons. First, we determined the multilocus genotype of each *S. paradoxus* forest isolate. Then, we assayed each *S. paradoxus* forest isolate for killer toxin production and investigated whether these killer yeasts selected for resistant *S. paradoxus* strains in their local environments. We tested for selection by toxins over time, expecting resistance after a killer strain was detected (but not before), and over space, expecting resistance close to a killer strain (but not far away), if these toxins selected for resistance (Figures 1a,b, 2a,b).

## 2 METHODS

### 2.1 *S. paradoxus* collection and identification from a forest floor environment

**FIGURE 3:**
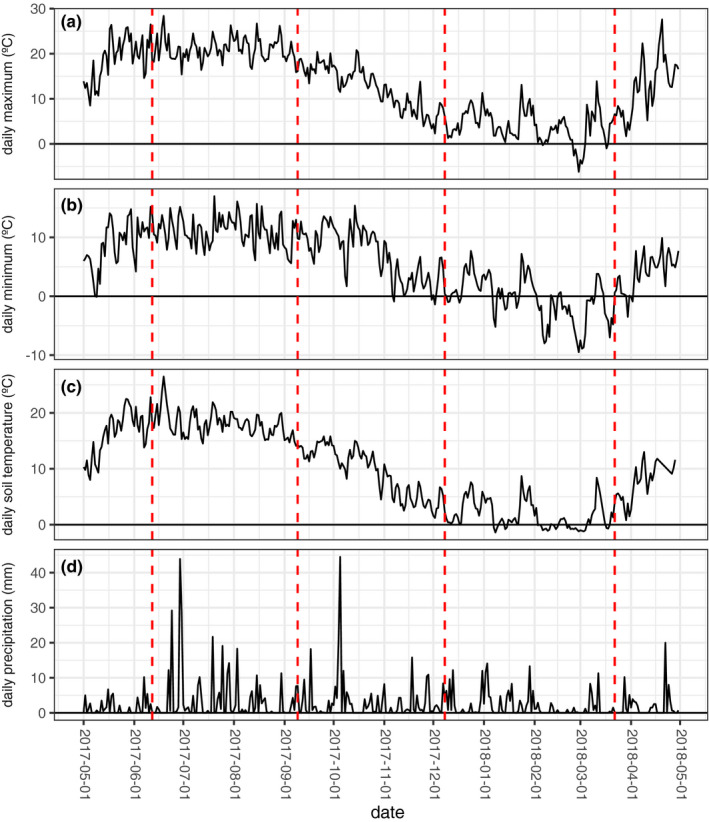
Temperature and precipitation over the sampling period. (a) Daily maximum temperatures at 2 m height. (b) Daily minimum temperatures at 2 m height. (c) Daily soil temperature measured at 5 cm depth. (d) Daily measured precipitation. Red dashed lines indicate sampling dates. Weather data are from the Dörnick weather station, 7 km from the sampled site (DWD Climate Data Center, 2018a, 2018b, 2019)

We collected *S. paradoxus* isolates from a northern German forest over four seasons in 2017–2018 (Figure 3). Strains were collected on 12 June 2017 (late spring), 9 September 2017 (late summer), 8 December 2017 (late fall), and 22 March 2018 (late winter) from soil and leaf litter next to eight oak trees in the Nehmtener Forst, Nehmten, Schleswig‐Holstein, Germany (approximate latitude 54.1, longitude 10.4) (Table 1). The trees were between 12 and 746 meters from one another. We collected strains using the direct plating protocol described previously (Boynton et al., 2019). Briefly, we collected samples of approximately 5 cm^3^ compressed leaf litter and soil from four locations evenly spaced around each tree base on north, south, east, and west sides of the tree. Each sample was collected within 1 m of the tree base, and repeated samplings from the same side of each tree were within approximately 50 cm of one another. We mixed each sample with 10 ml sterile water, vigorously vortexed the mixture, and spread 200 µl onto each of two solid selective plates (3 g yeast extract, 5 g peptone, 10 g sucrose, 3 g malt extract, 1 mg chloramphenicol, 80 ml ethanol, 5.2 ml 1 M HCl, and 20 g agar per liter) (Sniegowski et al., 2002). After 3 days, up to twelve yeast colonies with *Saccharomyces*‐like morphology (round, cream‐colored colonies) were randomly picked and stored in 20% glycerol at −70 to −80°C.

**TABLE 1:** Number of samples genotyped at each tree and date

Tree	June 2017	September 2017	December 2017	March 2018
Tree 1	7	10	5	10
Tree 2	5	10	5	10
Tree 3	5	6	5	5
Tree 4	7	10	6	10
Tree 6	0	1	0	0
Tree 7	10	10	10	10
Tree 8	9	1	5	6
Tree 9	5	6	3	0
Totals	48	54	39	51

We used morphological and mating screenings to determine whether yeast colonies were *S. paradoxus* as described previously (Boynton et al., 2019). Colonies were first screened for the ability to form *Saccharomyces*‐like asci (“tetrads”) on sporulation medium (20 g potassium acetate, 2.2 g yeast extract, 0.5 g dextrose, 870 mg complete amino acid mixture, and 25 g agar per liter). Colonies that produced tetrads were then mated with the *S. paradoxus* tester strain NCYC 3708. Because haploid spores from different *Saccharomyces* species can mate to form zygotes (Greig et al., 2002), this mating test only identifies isolates to genus. However, the vast majority (over 99%) of *Saccharomyces* isolates from the Nehmten forest were identified as *S. paradoxus* in a previous study (Boynton et al., 2019), and we therefore assumed that all isolates that successfully mated with NCYC 3708 were *S. paradoxus*.

We randomly selected up to five *S. paradoxus* isolates from each environmental combination of collection month, source tree, and source substrate (leaf litter or soil) for further genotype and phenotype tests. Some environmental combinations produced less than five isolates, and we included all available isolates for these combinations. In total, a collection of 192 isolates were used for genotyping, killer toxin production screening, and toxin resistance screening (Table 1).

### 2.2 Microsatellite genotyping

We genotyped all 192 *S. paradoxus* forest isolates plus 23 *S. paradoxus* isolates from the *Saccharomyces* Genotype Resequencing Project (SGRP) using microsatellite loci and protocols described previously (Babiker & Tautz, 2015; Boynton et al., 2019; Hardouin et al., 2015). SGRP strains were included to put local forest *S. paradoxus* into a population context and included 22 strains from the European *S. paradoxus* population and one from the Far Eastern *S. paradoxus* population, which we used as an out‐group (Liti et al., 2009). Lengths of nine microsatellite loci on six chromosomes were determined for all strains (Table 2) (Boynton et al., 2019). We amplified loci in 5 µl multiplex PCRs containing four or five primer pairs each; reactions were composed of one *S. paradoxus* colony, 2.5 µl Qiagen Multiplex PCR master mix, and 0.2 µM each primer in PCR‐grade water. Forward primers were labeled with FAM, HEX, or NED fluorophores at the 5’ end. PCR cycling, dilution, and denaturation were carried out using previously described protocols (Babiker & Tautz, 2015; Hardouin et al., 2015). We determined fragment lengths using an ABI 3730 DNA analyzer, Geneious 8.1.8 (https://www.geneious.com), and the Geneious microsatellite plugin 1.4.4, with GeneScan 500 ROX as a size standard, as previously described (Boynton et al., 2019).

**TABLE 2:** Microsatellite loci used to genotype forest *Saccharomyces paradoxus* isolates

Name	Chromosome	Repeat sequence	Fragment length (CBS432)	Forward primer	Reverse primer	Number of alleles (forest only)	Number of alleles (including SGRP)	*F*_is_
Chrom16.CTT	16	CTT	223	FAM‐CCTCATGGGTTTCGTCGTCT	CGGCTTTGGAATCCTGGACT	2	3	1
Chrom11.TTG	11	TGG	274	FAM‐ATCCTGCTTTGCGTCGAAGA	GCTAACTCCGCTACTCACCC	3	6	1
Chrom4.TAAA	4	TAAA	212	HEX‐CGGGGTTTTTCAATTCTTTGAAAAC	CGGCACTACCTATTTACCAAGTAAT	3	4	1
Chrom4.CAA	4	CAA, CAG	261	HEX‐GATCCACCATGGGACCACAA	AGCCATTGACTCTGCTTGCT	3	5	1
Chrom15.CAA	15	CAA, CAG	295	HEX‐TTGGGAATGGGCGCTACTTT	CCCGTGGAACAGCACCATAT	5	7	0.99
Chrom2.ATT	2	TAT	223	FAM‐GGTTAACCTGCCTGTTGAGGA	GCATCTCCGTCCTCCAAACA	6	9	1
Chrom6.CA	6	AC	184	HEX‐GCGGAGGGCTTATTCATCTT	CCTCGCTATATCCGTCTCGC	12	15	0.99
Chrom11.CAA	11	GCA, CAA, CAG	284	HEX‐GGTGCCTGAAGTGGAAAGGT	GCTCGCTGATGTTGTTCCTG	7	9	0.99
Chrom2.TAG	2	CTA, CAA, CAG	249	NED‐GCCAGGCCAGATAATCAGCA	ACCAGCCTGGATATGAGGGT	2	3	1

Note

Loci were previously described in Boynton et al. (2019).

### 2.3 Screening for killing ability

**FIGURE 4:**
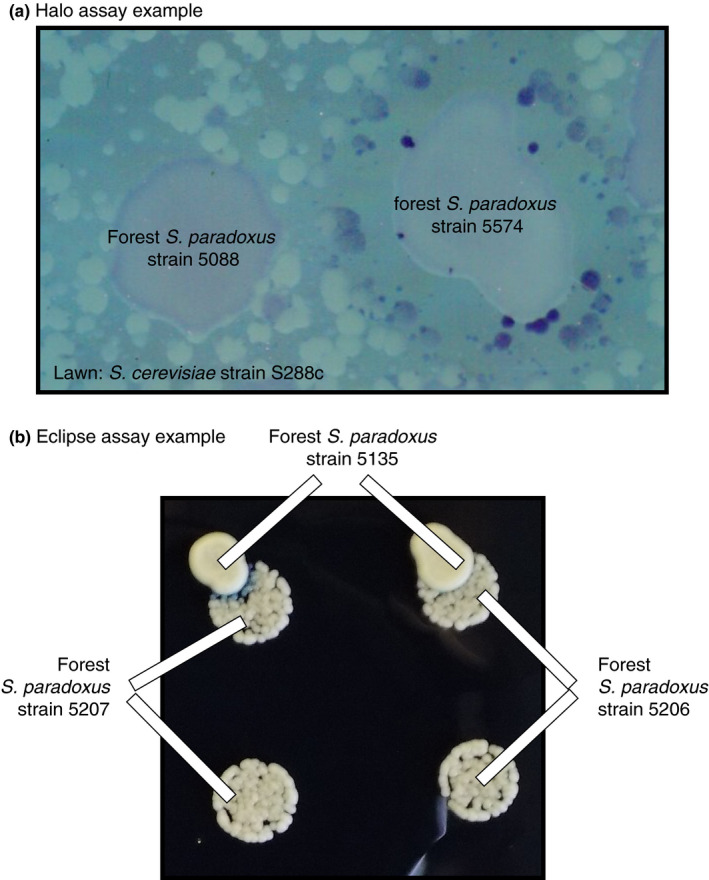
Examples of assays used to detect killer toxin activity and resistance to killer toxins. (a) Example of a “halo assay.” The forest *Saccharomyces paradoxus* strains 5088 and 5574 were dropped onto a lawn of the *Saccharomyces cerevisiae* tester strain S288C. Strain 5574 induced an empty zone in which strain S288C could not grow, ringed by S288C colonies containing dead cells that dyed blue with methylene blue. Strain 5088 produced no empty zone or blue S288C colonies. (b) Example of a “eclipse assay.” The top row is assay spots in which the forest killer *S. paradoxus* strain 5135 was dropped onto drops of the target forest *S. paradoxus* strains 5207 and 5206. The bottom row is control spots of 5207 and 5206. Strain 5135 induces a semicircle of small colonies that stain blue with methylene blue in strain 5207 but not 5206

Each of the 192 forest *S. paradoxus* strains was tested for the ability to kill five sensitive tester *S. cerevisiae* strains using “halo assays” (Boynton, 2019; Chang et al., 2015; Woods & Bevan, 1968). To conduct a halo assay, drops of liquid containing potential killer cells were pipetted onto a lawn of potentially sensitive cells; a zone of inhibition (a “halo”) was observed if a toxin produced by drop cells killed or inhibited lawn cells (Figure 4a). Tester *S. cerevisiae* strains included Y55, S288C, and a previously engineered sensitive strain derived from BY4741 and BY4742 (WS‐29‐10) (Wloch‐Salamon et al., 2008). We also tested whether forest *S. paradoxus* killed two isolates of a K2 killer strain (Wickner strain 1387) that had been cured of its toxin‐producing virus by incubation at 37 or 40°C (Wickner, 1974, 1980). Curing success was confirmed experimentally by performing a halo assay using the tester *S. cerevisiae* strain WS‐29‐10.

To perform halo assays, each tester *S. cerevisiae* strain was first evenly spread onto Killer Test Medium plates (10 g yeast extract, 20 g peptone, 20 g glucose, 15 g agar per liter, with 0.01%–0.03% methylene blue, adjusted to a pH of approximately 4.6 with citric‐phosphate buffer) at a final concentration of approximately 10^4^ cells per ml of medium. After tester *S. cerevisiae* lawns dried, each forest *S. paradoxus* isolate was spotted onto the tester lawn in a 4 µl drop containing approximately 8 × 10^5^ stationary‐phase *S. paradoxus* cells. As controls, we also included drops of *S. cerevisiae* killers K1, K2, and K28 on each plate. Plates were incubated at 23°C for 4–6 days. We scored each *S. paradoxus* drop for a surrounding halo in which the tester *S. cerevisiae* strain did not grow. Often, dead *S. cerevisiae* cells at the edge of the halo appeared dark blue as a result of staining with methylene blue. Each test was conducted twice, and a forest *S. paradoxus* strain was scored as a “killer” if a halo was observed on at least one of the *S. cerevisiae* tester strains for at least one of the two tests.

### 2.4 Screening for evolution of killer resistance using eclipse assays

To understand whether toxin‐producing strains select for resistance to toxins in nature, we tested “target” nonkiller isolates, found at the same location or time as a killer strain, for sensitivity or resistance to toxins produced by the killer strain (Figures 1a, 2a). For each killer isolate identified in the screen for killing ability, we assigned two collections of target (i.e., potentially sensitive or resistant) forest *S. paradoxus* isolates from the full collection of 192 *S. paradoxus* isolates: a *temporal* collection and a *spatial* collection. The *temporal* collection of target isolates was composed of all strains collected at the same location (the same side of the same tree) as the killer at all available timepoints (Figure 1a). Because killers were discovered from all four timepoints, in some cases the collection of target isolates for a particular killer strain included only isolates found before or only isolates found after the killer, but all temporal collections included target isolates from at least three timepoints. The *spatial* collection of target isolates was composed of all isolates collected from the same tree and date as the killer strain, including isolates collected from the same side of the tree and other sides of the tree, plus five randomly chosen isolates from other trees collected on the same date as the killer (Figure 2a). For each killer strain, we assayed the sensitivity or resistance of every target strain in the killer's temporal and spatial collection.

We assayed each target forest *S. paradoxus* isolate for resistance to its assigned forest *S. paradoxus* killer using “eclipse assays” (Figure 4b) (Kishida et al., 1996). Eclipse assays are similar to halo assays, except that the killer spot is dropped onto a small spot of the target *S. paradoxus* isolate instead of a lawn of tester *S. cerevisiae* cells. Positive eclipse assays visually resemble partial solar eclipses, with the presence of an empty or blue semicircle indicating a killing reaction (Figure 4b). Eclipse assays were carried out on “eclipse media” at two different pH values (10 g yeast extract, 20 g peptone, 20 g glucose, 25 g agar, and 0.008% methylene blue per liter, adjusted to a pH of 4.1 or 3.6 with 12 ml or 16 ml 1 M citric acid per liter, respectively). Each test for resistance was carried out twice, once at each pH. Approximately 100 cells of each target isolate were spotted onto each eclipse medium plate in a volume of 5 µl. After spots had dried, each spot was overlaid with approximately 10^6^ killer forest *S. paradoxus* cells in a volume of 0.5 µl. Each plate also contained control target isolate spots which were not overlaid with a killer drop. Plates were incubated at 13°C for 6 days before being scored for evidence of killer toxin inhibition. We scored target strains as sensitive to a particular killer if it showed a positive reaction (zone of blue or dead cells) on either of the two tested media (pH = 4.1 or 3.6) and resistant if no positive reaction was observed.

### 2.5 Data analysis

**FIGURE 5:**
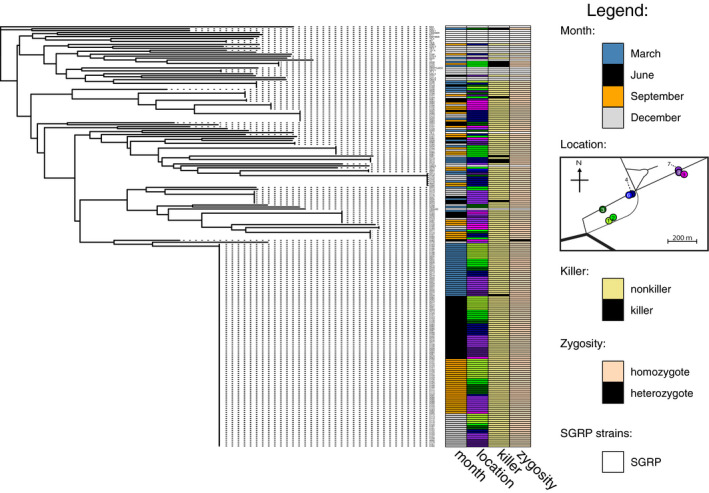
Neighbor‐joining tree of all 192 forest and 23 SGRP strains genotyped, with information on the month sampled, location, ability to kill tester *Saccharomyces cerevisiae*, and zygosity of each forest strain depicted with colored bars to the right of the tree. Information is not provided for SGRP strains, which are represented with white bars

A neighbor‐joining tree of microsatellite peak data was produced from Edwards distances using the adegenet 2.1.1 and poppr 2.8.3 packages in R 3.6.0 (Edwards, 1971; Jombart, 2008; Jombart & Ahmed, 2011; Kamvar et al., 2014, 2015; R Core Team, 2019). We tested for correlations between genetic distance among strains and difference in isolation time, and correlations between genetic and geographic distances, using Mantel tests in vegan 2.5‐6 (Oksanen et al., 2019). We used an analysis of molecular variance (AMOVA) to further determine the hierarchical spatial structure of genetic variation associated with individual trees, among trees, and among three clusters of trees (southwest, middle, and northeast, see inset in Figure 5). The AMOVA was conducted using the R packages poppr 2.8.3 and ade4 1.7‐13 (Dray & Dufour, 2007), and significance was determined by permuting the dataset 999 times. We also used binomial tests to determine whether the number of killer strains associated with a particular tree or timepoint was different from that expected by random chance. Because we performed multiple tests for temporal and spatial structure using the same set of genetic data, we adjusted p‐values for these tests and the tests for linkage disequilibrium below using Holm's adjustment (Holm, 1979). Expected heterozygosities of the forest population and the European SGRP strains were calculated using adegenet 2.1.1. We visualized the phylogenetic tree using the ggplot2 3.2.1 and ggtree 1.16.6 libraries (Wickham, 2016; Yu et al., 2017, 2018). The relationship between sampling effort and the number of unique genotypes observed, including a 95% confidence interval, was visualized using the interpolation method described by Chao et al. (2014) with 50 bootstrap samplings, using iNEXT 2.0.19 (Hsieh et al., 2019).

Linkage disequilibrium was calculated using the Index of Association (*I_a_*), determined by comparing associations between alleles according to the following equation: *I_a_* = (*V_o_*/*V_e_*) − 1 (Smith et al., 1993), where *V_o_* is the observed variance in number of loci at which pairs of individuals in the dataset differ and *V_e_* is the expected variance in the number of loci at which pairs of individuals in the dataset differ. An *I_a_* of zero indicates no association among loci, and *I_a_* values significantly higher than zero indicate nonrandom associations among loci (i.e., some loci appear together in the same genome more often than expected by chance) (Smith et al., 1993). Significance was determined by shuffling alleles at each locus 999 times while preserving heterozygosity and allelic structure. We calculated *I_a_* for the entire forest *S. paradoxus* dataset (no correction for clonality) and for the dataset containing one example of each unique genotype (correction for clonality) using poppr. *F*_is_ was calculated at each locus using the pegas 0.11 package in R (Paradis, 2010).

We modeled the target strains’ resistances to killer toxins as functions of distance in time or space between when and where target and killer strains were found (Figures 1, 2). We produced separate mixed‐effects logistic regressions for the temporal and spatial datasets. The response variable for both regressions was whether or not a target *S. paradoxus* strain was resistant to its killer. The fixed predictor variables were difference in time between when the killer and target strains were found for the temporal dataset and the categorical difference in location between where the killer and target strains were found (i.e., same side of the same tree, different side of the same tree, or different trees) for the spatial dataset. Random effects for both regressions were the identities of the killer and target strains because not all killers were tested against all target strains. Models were constructed and tested in R using the lme4 1.1‐21 and car 3.0‐3 packages (Bates et al., 2015; Fox & Weisberg, 2019).

## 3 RESULTS

### 3.1 Stable *S. paradoxus* diversity over space and time

**FIGURE 6:**
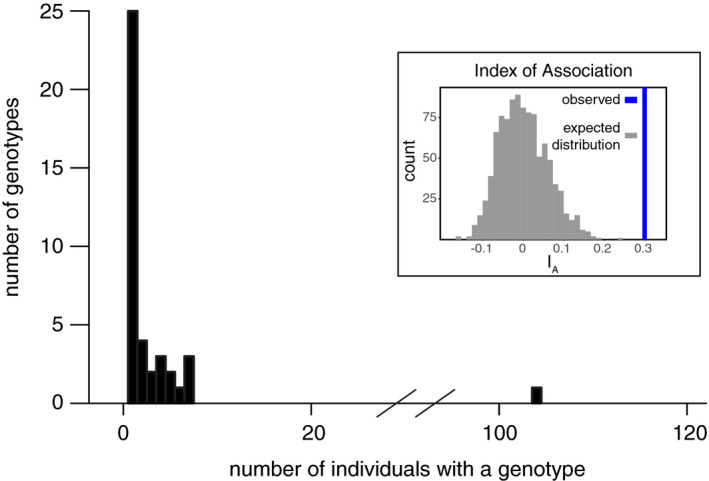
Histogram of unique observed genotypes. The *x*‐axis depicts the number of strains in which a genotype was observed, and the *y*‐axis depicts the number of genotypes with a given number of observed strains. The inset depicts the Index of Association (*I_a_*) of the clone‐corrected population (i.e., each genotype is represented only once in the Index of Association calculation). The thick blue line represents the calculated *I_a_* for the population, and the gray bars represent the distribution of expected *I_a_* for a population with the same allele frequencies, but randomly shuffled

**FIGURE 7:**
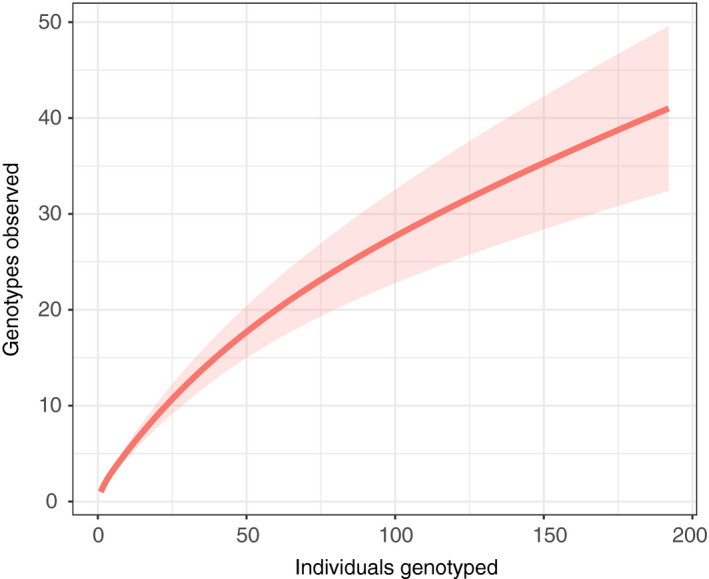
The relationship between sampling effort (number of individuals genotyped) and the number of unique genotypes observed. The thick line represents average genotypes observed as a function of isolates sampled, and shaded areas represent 95% standard errors. The curve was calculated using the iNEXT package in R and the interpolation method described by Chao et al. (2014), with the shaded area representing a 95% confidence error calculated with 50 replications

**FIGURE 8:**
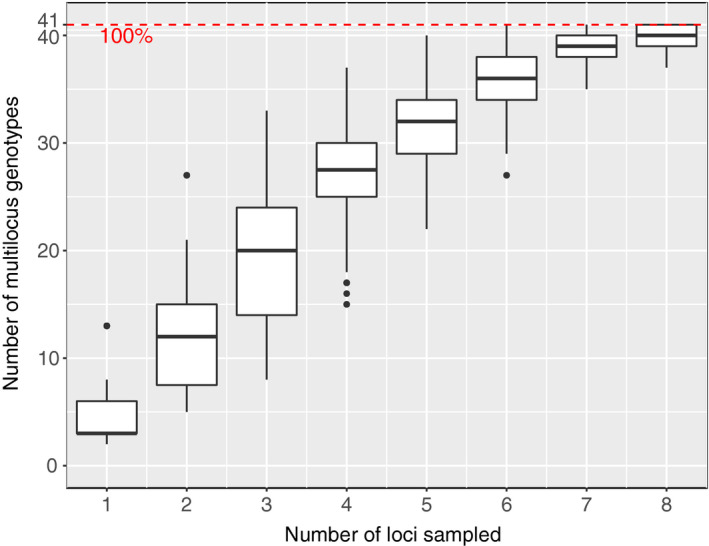
The relationship between number of microsatellite loci sampled and the total number of unique genotypes observed. Box plots indicate the medians, quartiles, and ranges of observed genotypes after randomly sampling 100 times. The dotted red line is the total number of genotypes (41) observed when measuring all nine loci

There were 41 unique genotypes among the 192 forest *S. paradoxus* isolates (Figures 5, 6). However, our sampling did not saturate *S. paradoxus* diversity, as indicated by an increasing relationship between analyzed isolates and observed unique genotypes (Figure 7), and there is further unsampled diversity in the forest. Among the sampled forest isolates, sixteen genotypes were represented by multiple individuals, and no forest *S. paradoxus* isolate had the same genotype as a reference SGRP individual. The number of alleles per locus ranged from 2 to 12 (forest isolates only) or 3 to 15 (all isolates) (Table 2). We assume all individuals sharing a multilocus genotype were clones of one another because the relationship between number of loci sampled and unique genotypes detected plateaued between eight and nine loci (Figure 8). Over half of the forest *S. paradoxus* isolates (104) were members of a single genotype, and we assume these were all clones of one another (Figure 6); this common genotype was present at all four sampling times and next to seven of the eight sampled trees (Figure 5). The expected heterozygosity, a measure of genetic diversity, of the forest *S. paradoxus* isolates was 0.41, which was less than the expected heterozygosity of measured European SGRP strains (0.60). Ten of the 192 forest strains (5%) produced a toxin that killed at least one tester *S. cerevisiae* strain. The number of tester *S. cerevisiae* strains killed ranged from one (three forest *S. paradoxus* isolates) to all five (one isolate). This incidence of forest killer isolates is an underestimate because only killer strains that killed a tester strain under the pH and temperature conditions of our screen could be detected. Systematic tests with more tester strains and a variety of pH and temperature values are likely to uncover more killer isolates.

In general, genetic differences in the forest *S. paradoxus* population did not correlate with time or space, although individual clones were somewhat spatially restricted. There was no correlation between genetic distance and temporal distance (Mantel's *r* = −0.03, adjusted *p* = 1.0) or spatial distance (Mantel's *r* = −0.003, adjusted *p* = 1.0) (Figure 5). However, there was some spatial structure within and among individual tree islands (Table 3): 86.4% of genetic variation was found to be associated with individual trees (less than expected by random chance, adjusted *p* = 0.009), 12.5% among trees (more than expected by random chance, adjusted *p* = 0.009), and 1.1% among clusters of trees (not significantly different from random chance, adjusted *p* = 1.0). We attribute this pattern to the strong clonal nature of the population, limited dispersal of some *S. paradoxus* clones, and specifically to the frequent observation of clones restricted to a single tree. The presence of a clone restricted on a single tree will both decrease the genetic variation present at that tree, compared to the entire population, and increase genetic heterogeneity among trees. Of the 16 clones found more than once, eight were restricted to a single tree and eight were present on multiple trees. The largest clone restricted to a single tree included seven isolates (Figure 5). Of the ten detected killer *S. paradoxus* isolates, eight were found during cold months (December and March), a frequency not different from random chance (binomial test of eight successes in 10 trials compared to 0.47, the proportion of total December and March isolates, adjusted *p* = 1.0) (Figure 5). Killer isolates were found at five of the eight sampled locations, with five isolates found on Tree 2 (three of these five were clones with the same microsatellite genotype). The number of isolates on Tree 2 was not more than expected by random chance (binomial test of five successes in 10 trials compared to 0.16, the proportion of total strains found on Tree 2, adjusted *p* = 0.06).

**TABLE 3:** Analysis of molecular variance (AMOVA) for microsatellite genotypes

Source of variation	Df	Sum of squares	Mean squares	Sigma	Percent of total variance	Variance significance*
Within tree	184	601.02	3.27	3.27	86.43	Less than expected by random chance, adjusted *p* = 0.009
Among trees within clusters	5	64.19	12.84	0.47	12.46	More than expected by random chance, adjusted *p* = 0.009
Among tree clusters	2	40.18	20.09	0.04	1.11	Not different from random chance, adjusted *p* = 1.0
Total	191	705.39	3.69	3.78	100	

* Significance was calculated by comparing against a distribution of 999 permutations of the data.

In addition to its high clonality, the forest *S. paradoxus* population was characterized by high inbreeding. Only one out of the 192 isolates was heterozygous, at three of the nine loci (Figure 5; Table 2). The forest *S. paradoxus* population also had high linkage disequilibrium, as indicated by a high index of association among alleles: After correcting for clonality, the index of association among forest isolates was significantly different from zero (*I_a_* = 0.30, adjusted *p* = 0.009) (Figure 6 inset). Without correcting for clonality, *I_a_* was 2.87 (adjusted *p* = 0.009).

### 3.2 No evidence for selection for toxin resistance

As with microsatellite genotypes, there was diversity among forest strains’ responses to killer toxins, but no evidence for selection for resistance over time or space. Resistant strains were just as common after as before the date a killer strain was found (*Χ* ^2^ = 1.20, *df* = 1, *p* = 0.27) (Figure 1c). Similarly, resistant strains were just as common close to as far away from killer strains (*Χ* ^2^ = 1.78, *df* = 2, *p* = 0.41) (Figure 2c). Of the 206 total killer–target combinations tested, 59 (29%) resulted in killer inhibition of the target yeast. In agreement with previous observations of an optimal pH of approximately 4.0–4.8 for well‐known *S. cerevisiae* killer toxins (McBride et al., 2008; Woods & Bevan, 1968), inhibition was more frequent on high‐pH (pH = 4.1) than low‐pH (pH = 3.6) media: 58 killer–target combinations showed inhibition on high‐pH media, compared to 27 on low‐pH media. Additionally, and anecdotally, some killer yeasts were more effective against the tested target strains than others. Of the ten killer isolates, three produced effective toxins against 75% or more of tested target strains, while the other seven produced effective toxins against less than 9% of tested target strains. However, our sampling was not designed to systematically test for this effect (i.e., each individual killer was tested against a different set of target strains), and it is not possible to tell from these data whether the difference in apparent killing ability is an effect of killer or target strains.

## 4 DISCUSSION

### 4.1 The forest *S. paradoxus* population is surprisingly robust to seasonal environmental changes

We found no evidence that seasonal‐scale environmental changes select for seasonally adapted individuals from the diverse forest *S. paradoxus* population. Although we identified 41 unique *S. paradoxus* microsatellite genotypes, the genotype of an individual did not correlate with the season in which it was found. We expected potentially selected loci to be linked to our measured microsatellite loci because the *S. paradoxus* population experiences low recombination and high clonality (Figures 5, 6 inset). Similar to genotype diversity, monthly‐scale selection was not associated with killer toxin‐related phenotypes. *Saccharomyces paradoxus* population stability is consistent with a lack of local adaptation to substrate previously observed in the same population and with persistent North American *S. paradoxus* genomes observed in a previous study (Boynton et al., 2017; Xia et al., 2017). However, these results contrast with observations of fitness diversity among North American *S. paradoxus* and with changes in individuals’ fitness ranks after environmental perturbation (Bleuven et al., 2020).

The presence of a single clone encompassing 104 out of the 192 sampled isolates is an extreme example of the observed population stability. This single clone was common at all sampled timepoints and present on seven of the eight sampled trees. Surprisingly, we detected killer toxin secretion in one of these 104 individuals, but not in the other 103 (Figure 5). This observation is surprising because we expect killer toxins to be coded on vertically transmitted cytoplasmic dsRNA viruses in wild *S. paradoxus*. We have several potential explanations. First, rare horizontal transmission of the dsRNA viruses coding for killer toxins may be possible in nature. While these viruses are generally assumed to be exclusively transmitted vertically (Boynton, 2019; Pearson et al., 2009), researchers have successfully transfected *S. cerevisiae* with dsRNA viruses in laboratory environments (El‐Sherbeini & Bostian, 1987; Schmitt & Breinig, 2002). Additionally, the phylogenetic history of some *S. paradoxus* viruses is consistent with horizontal transmission from *S. cerevisiae* or vice versa (Fredericks et al., 2021). It is plausible that viruses from dead or lysed cells may have transformed nearby living *S. paradoxus*. Second, the toxin‐encoding virus may have been present in an ancestor of the common clone, but have been cured in a later ancestor of most individuals. Killer viruses are easily cured in laboratory *S. cerevisiae* isolates at temperatures of 37°C (Wickner, 1974), and we might expect rare incidences of curing *S. paradoxus*, which has a lower optimal growth temperature than *S. cerevisiae* (Robinson et al., 2016), on the hottest of summer days in northern Germany (Figure 3). Finally, the entire clone may produce a killer toxin, but our assay was only sensitive enough to detect the toxin in one of the 104 individuals. Killer toxins are only effective under a narrow set of environmental conditions, including pH and temperature (Woods & Bevan, 1968). The detected killer was only effective in one replicate test against sensitive strain WS‐29‐10. It is possible that slight variation in growth media for this particular test, or variation in incubator temperature, led to development of a halo in this test but not others.

The forest *S. paradoxus* population's stability contrasts with observed rapid adaptation in laboratory‐selected yeast populations. *Saccharomyces* yeast populations, including *S. paradoxus* and its sister species, the laboratory model *S. cerevisiae*, have adapted over days to months to changes in temperature (Huang et al., 2018), nutrient availability (Goddard & Bradford, 2003), environmental stresses (Dettman et al., 2007), and exposure to killer toxins (Pieczynska et al., 2016). The difference in adaptation dynamics between forest and laboratory *S. paradoxus* populations is unlikely to be due to the number of cells in the population (although it may be influenced by differences in generation times between laboratory and forest populations) because forest *S. paradoxus* populations have more cells than many laboratory populations: Multiplying tens to thousands of estimated *S. paradoxus* cells per gram of leaf litter (Kowallik & Greig, 2016) by metric tons of oak leaf litter per hectare per year in temperate forests (Bray & Gorham, 1964) gives 10^7^–10^9^ *S. paradoxus* cells per hectare of forest. We found some evidence for dispersal limitation (Table 3), and some individual clones associated with individual trees. But the overall population was well mixed over the 700 m forest transect, as indicated by low genetic variation among tree clusters (Table 3). In particular, the most common *S. paradoxus* clone was distributed throughout the sampled area (Figure 5) suggesting that the dispersal limitation over tens of hectares in this forest is minor.

Nonetheless, laboratory *Saccharomyces* populations are often under selective pressures outside of the range of the environmental conditions *Saccharomyces* experiences in nature. When studying laboratory adaptation, researchers generally purposely choose selective environments that are very different from standard laboratory growth conditions (Swamy & Zhou, 2019); these selective environments are also likely to be outside of the range of environmental conditions experienced by wild yeasts. Additionally, density may be especially important for laboratory rapid adaptation. Forest *S. paradoxus* populations are considerably less dense than many adapting laboratory populations, with up to thousands of cells per gram of soil instead of as many as 10^6^ cells per ml of laboratory media (Kowallik & Greig, 2016; Zeyl et al., 2003). Adaptation to high density can confound observations of rapid adaptation to new environmental conditions during experimental evolution.

The nature of selective pressures on *S. paradoxus* in the forest is poorly understood compared to those in laboratory populations. Forest selective pressures are almost certainly more complex than selective pressures in laboratory experimental evolution: Evolution experiments generally manipulate one or very few selective pressures in an otherwise completely controlled environment. In the forest, selective pressures are likely to be varied and could even be conflicting. Our sampling was designed to detect selection on a seasonal timescale, but it is possible that selection is instead operating on shorter or longer timescales. For example, our experimental design might not detect selection during bursts of growth following rain events (Anderson et al., 2018). We also did find some seasonally restricted clones: Three clones (out of the sixteen clones that were represented by more than one individual), represented by 2–3 individuals each, were only found at one timepoint, and two of these three were restricted to a single tree. These three clones may indeed be locally adapted, either seasonally or spatially, but future sampling and phenotypic assays are needed to determine whether they are truly temporally restricted and whether they do indeed grow faster in seasonal environments. Finally, selection may operate differently on dormant cells or spores than on actively reproducing cells. We do not know how much time *S. paradoxus* spends in dormant or active states in the forest, but previous researchers have estimated the relative numbers of meiotic (resulting in dormant spores) to mitotic (resulting in active cells) cell divisions in wild *S. paradoxus* to be 1:1,000, based on genomic data (Tsai et al., 2008). Because our sampling strategy was equally likely to pick up spores and actively growing cells, we may have inadvertently compared killer and potentially sensitive strains that were not interacting with one another and had not had the opportunity to select on each other. However, our observation of generally low incidences of sensitivity across tested isolates stands regardless of whether isolates were active or dormant at the time of isolation. Overall, our observational strategy would not be able to detect the effects of weak selection in the forest, but we would expect to detect strong selective sweeps in response to temperature changes if they occurred.

### 4.2 Population stability suggests flexibility in a seasonally changing environment

The observed *S. paradoxus* stability in the face of seasonal changes may be the result of generalism, evolved over thousands of years of evolutionary time. We consider generalists to include individuals that experience environmental changes, but are not subject to selection by these changes because they are already adapted to the changing environment. European *S. paradoxus* diverged from East Asian *S. paradoxus* tens of thousands to hundreds of thousands of years ago (Liti et al., 2006), and *S. paradoxus* could have been living in northern Germany as early as the end of the last glaciation, approximately 18–19,000 years ago (Stroeven et al., 2016). *Saccharomyces paradoxus* most likely adapted to seasonal fluctuations during this time and may be phenotypically plastic with regard to the biotic and abiotic variation it normally encounters over the course of months. Alternatively, *S. paradoxus* may be so static, and have so little genetic variation, that seasonal changes occur too quickly to impose effective selection on the population. While this scenario is possible, we consider it unlikely because a previous study showed rapid spikes and declines in a *S. paradoxus* population as a result of rain events, environmental perturbations more frequent than seasonal changes, in a similar forest habitat (Anderson et al., 2018).

The suggestion of past adaptation is further supported by high observed resistance to naturally occurring killer toxins (Figures 1c, 2c). Observations of infrequent killing in killer–target pairings suggest that sensitive *S. paradoxus* adapted to the presence of toxin‐producing *S. paradoxus* many generations ago. If resistance is widespread, the viruses that encode killer toxins may still be maintained in the population if they do not impose fitness costs or if they behave as selfish genetic elements (Boynton, 2019; Kast et al., 2015). The laboratory evidence for costs of hosting killer toxin‐producing viruses is mixed and depends on the yeast species investigated and investigation method used (Pieczynska et al., 2017; Wloch‐Salamon et al., 2008). Alternatively, toxins may simply not be effective in the soil environment. To be effective, toxin molecules must reach sensitive cells through diffusion in soil, and the environmental temperature and pH must be in the narrow range required for the toxin to be active (Lukša et al., 2016; Woods & Bevan, 1968). If toxins are ineffective in nature, however, we would expect resistance to be uncommon among natural isolates, instead of common (resistant strains were observed in 71% of tested killer–target combinations). Further research is needed to investigate the action of killer toxins in nature and the ecological and evolutionary mechanisms maintaining killer viruses in forest populations.

High clonality and inbreeding support our observations of a stable forest *S. paradoxus* population. Sex is predicted to evolve, and outcrossing to be favored, in environments with coevolutionary selection pressures (Morran et al., 2011). The lack of observed recombination among forest *S. paradoxus* suggests that killer and sensitive *S. paradoxus* are not coevolving in the forest. Conversely, sex and outcrossing are not favored in stable environments because recombination breaks up locally adapted gene combinations (Barton & Charlesworth, 1998). The observed levels of clonality and inbreeding in the Nehmtener forest are consistent with observations of other European and North American *S. paradoxus* populations (Johnson et al., 2004; Koufopanou et al., 2006; Tsai et al., 2008; Xia et al., 2017). Wild European *S. paradoxus* are estimated to outcross once in every 100 meiosis events, and meiosis expected to occur only once in every 1,000 cell divisions (Tsai et al., 2008). Alternatively, outcrossing may be low in forest environments because *S. paradoxus* density is low in the forest and unrelated mates might be difficult to find. While the lack of observed *S. paradoxus* recombination is consistent with our other results suggesting that forest environments are stable from the point of view of generalist *S. paradoxus* individuals, more research, including population whole genome sequencing, is needed to fully understand the evolutionary reasons for low outcrossing in nature.

While *S. paradoxus* populations are robust to environmental changes on monthly timescales, our observations do not reject selection on shorter or longer timescales. As previously mentioned, rain events associated with increases in *S. paradoxus* populations may impose selective pressures on timescales shorter than our sampling scheme (Anderson et al., 2018). In the other direction, there is strong evidence that *Saccharomyces* species have been adapting to changing environments over longer timescales. For example, *S. cerevisiae* domestication over hundreds to thousands of years has led to changes in copy number variation, ploidy, and aneuploidy during adaptation to diverse environments (Peter et al., 2018). Additionally, partially reproductively isolated *S. paradoxus* populations are locally adapted to climactic conditions in North America (Leducq et al., 2014). The temporal scales at which selection is most effective are emerging as an important question for wild microbial populations.

### 4.3 The future of local *S. paradoxus* populations

An open question about microbial populations is how they will respond to anthropogenic climate change (Antwis et al., 2017), and our data suggest that European *S. paradoxus* populations will persist in the face of some forms of climate change. As long as temperatures remain within current seasonal ranges (i.e., maximum summer temperatures remain between 18 and 31°C) (Robinson et al., 2016), and the trees with which they associate remain in their current location, we expect *S. paradoxus* to survive, even as climate variance is predicted to increase (Vasseur et al., 2014). Similarly, we expect *S. paradoxus* populations to be robust to changes in seasonal timing, such as earlier spring temperatures or later winter temperatures (Cleland et al., 2007). Once mean or maximum temperatures increase, however, *S. paradoxus* may be replaced by related warm‐adapted yeasts. Specifically, *S. cerevisiae* is likely to replace *S. paradoxus* if maximum summer temperatures increase to be consistently above 31°C because the ranges of both yeasts are limited by maximum summer temperatures (Robinson et al., 2016). This possibility demonstrates how ecological processes can be as important as evolutionary processes in determining population and community responses to climate change.

## 5 CONCLUSIONS

The forest *S. paradoxus* population presents a paradox: It is characterized by stability, high dispersal, clonality, and inbreeding, while still including genetic and phenotypic diversity. In laboratory evolution, rapid adaptation can produce such diversity, but the evolutionary processes at play in the laboratory and forest are different. Our observations suggest that forest *S. paradoxus* do not repeatedly adapt to seasonal‐scale selective pressures and are instead generalists. Future research is needed to unite the natural history of these wild microorganisms with physiological and genetic studies of plastic responses to environmental changes. *Saccharomyces paradoxus* is an exciting opportunity to explore connections between forest and laboratory biology and to understand how the biology of a model microbe influences its ecology and evolution—including its stability—in its native habitat.

## CONFLICT OF INTEREST

The authors declare no competing interests.

## AUTHOR CONTRIBUTIONS

**Primrose J. Boynton:** Conceptualization (lead); Data curation (lead); Formal analysis (lead); Investigation (lead); Methodology (lead); Project administration (lead); Resources (lead); Validation (lead); Visualization (lead); Writing‐original draft (lead). **Dominika Wloch‐Salamon:** Formal analysis (supporting); Investigation (supporting); Methodology (supporting); Writing‐review & editing (supporting). **Doreen Landermann:** Investigation (supporting); Resources (supporting); Writing‐review & editing (supporting). **Eva H. Stukenbrock:** Conceptualization (supporting); Funding acquisition (lead); Investigation (supporting); Project administration (lead); Resources (supporting); Writing‐review & editing (supporting).

## Data Availability

Data for this project are available from the Edmond Open Access Data Repository (https://dx.doi.org/10.17617/3.3u). Deposited datasets include a list of *Saccharomyces paradoxus* isolates with sampling data, microsatellite data for each isolate, the ability of each isolate to inhibit each tester *Saccharomyces cerevisiae*, and the ability of killer *S. paradoxus* to inhibit tester *S. paradoxus* in temporal and spatial assays for killer resistance.
